# Bioelectrical impedance phase angle in sport: a systematic review

**DOI:** 10.1186/s12970-019-0319-2

**Published:** 2019-11-06

**Authors:** Olivia Di Vincenzo, Maurizio Marra, Luca Scalfi

**Affiliations:** 1 Department of Clinical Medicine and Surgery, Federico II University Hospital, Via S. Pansini 5, 80138 Naples, Italy; 2 Department of Public Health, School of Medicine, Federico II University, Naples, Italy

**Keywords:** Phase angle, Bioimpedance analysis, Athletes, Sport, Physical activity

## Abstract

**Background:**

Phase angle (PhA) is a raw BIA variable that has been gaining attention in recent years because it is supposed to be an index of the ratio between extracellular and intracellular water, body cell mass, and cellular integrity.

The aim of this systematic review was to evaluate the variability of PhA between different sports and its relationships with sport performance. Additionally, we investigated whether PhA depends on gender or age, and analyzed the differences between athletes and controls.

**Methods:**

A systematic research using PubMed, Scopus and Web of Science up to June 2019 was performed. Selection criteria included studies on subjects who practice sports in planned and continuous modality at competitive or elite level.

**Results:**

Thirty-five papers met the inclusion criteria (twenty-one cross-sectional data, fourteen longitudinal data). A few but convincing studies have shown that mean PhA is higher in athletes vs. controls. PhA increases with age and is likely to be higher in male than female athletes. A large variability in PhA is observed for the same sport, while it is still uncertain to what extent PhA differs between various sports. There are no clear relationships of PhA with sport performance or training/untraining.

**Conclusion:**

It is still to be defined to what extent PhA varies between different sports and changes with training/untraining. It can be argued that for a given sport much more data should be collected in a systematic way and for a period of time appropriate in order to determine changes and trends. This is even more crucial in the case of intervention studies.

## Background

Bioelectrical impedance analysis (BIA) is a widely used, non-invasive field method for assessing body composition, which measures the electrical characteristics of human body either at 50 kHz (single-frequency BIA) or at several frequencies in the range 1–1000 kHz (multifrequency BIA and BIS = bioimpedance spectroscopy). Impedance (Z) is the opposition of the body to an alternating current, resulting from resistance (R) to the current that flows through tissue containing water plus electrolytes, and reactance (Xc), which is associated with the capacitive component of tissues (e.g. cell membranes and tissue interfaces) [1]. In addition, phase angle (PhA), which is also stated as the arctangent of the Xc to R ratio, describes the angular shift (phase difference) between voltage and current sinusoidal waveforms; in humans the current reaches at regular intervals its maximum/minimum peaks after the voltage (positive PhA values) and this lag is most likely due to cell membranes and tissue interfaces [1, 2].

Using BIA, total body water (TBW) and fat-free mass (FFM) can be estimated by means of predictive equations, which include BIA variables and almost always variables such as age, stature and weight. Alternatively, directly-measured raw BIA variables, such as PhA at 50 kHz or impedance ratio (IR = the ratio between Z at higher frequencies and Z al lower frequencies), have been gaining attention because they are considered indexes of water distribution (ratio between extracellular water-ECW and intracellular water-ICW), body cell mass (BCM), and cellular integrity [2]. PhA and IR have been shown to be significantly associated with muscle strength and physical activity [3, 4] and to vary between gender and with aging [5, 6] in line with that is known about physiological changes in BCM and ECW/ICW.

In sport science the assessment of body composition has different applications such as identifying individual’s characteristics critical to performance, evaluating the effects of training programs, managing weight strategies in weight-category sports, etc. In this regard, BIA has been used in athletes as a field technique for estimating TBW and FFM. Indeed, there is still limited research and it is uncertain to what accuracy BIA may be used in athletes for single measurements or for tracking body composition changes [7]. Even less attention has been paid to raw BIA data. A recent review has shown that Bioelectrical Impedance Vector Analysis (BIVA) of both R and Xc has yielded some conflicting results on the use if BIA for identifying dehydration [8, 9]. On the other hand, at least in theory, the use of PhA or IR may be crucial in evaluating athletes’ body composition because it can provide useful data on the percentage of BCM in FFM (structural muscle quality) in both cross-sectional and longitudinal studies. A recent paper [10] supported this view showing in 202 athletes that PhA significantly correlated with ICW and the ICW/ECW ratio. In this context, the purpose of this systematic review was to evaluate the variability of PhA among athletes and its relationship with sports performance. Additionally, we wanted to investigate whether PhA differs between athletes and controls or between different sports.

## Methods

### Search strategy

Two authors (ODV and MM) independently performed a literature search up to June 2019 of the electronic databases PubMed, Scopus, and Web of Science.

The following terms were used as search strategy string: (“bioelectrical impedance” OR “bioimpedance” OR BIA) AND “phase angle” AND (spor* OR athlet* OR “physical activity” OR fitness OR train*).

The Preferred Reporting Items for Systematic Reviews and Meta-Analyses (PRISMA) [11] were followed for performing the present review. Due to the study type (systematic review), ethical approval was not necessary according to local registration.

### Eligibility criteria

The PICOS strategy was defined as follows: “P” (patients) corresponded to participants of any age, sex or ethnicity, “I” (intervention) designated regular physical exercise at amateur, elite and professional level, “C” (comparison) indicated no physical exercise or low physical activity, “O” (outcome) corresponded to PhA, and “S” (study design) indicated cross-sectional or longitudinal studies.

The following eligibility criteria were applied: a) studies on athletes following exercise programmes with or without a control group; b) papers published from inception to June 2019; c) full papers published in peer-reviewed journals or in relevant congress proceedings; d) studies evaluating body composition using BIA phase-sensitive devices and yielding overt data on PhA; e) studies written in English. No restriction was applied to age of participants and sample size.

Studies with the following criteria were excluded: a) non-healthy athletes; b) articles without full-text availability, opinion pieces, review articles and editorials.

### Study selection and data extraction

Titles and abstracts from the electronic searches were screened independently by two authors (ODV and MM). The full texts of selected articles were checked by the same two authors to consider the fit with eligibility criteria. A third reviewer (LS) revised any differences in opinion to make a final decision.

An electronic database was designed to store all relevant data. Data were extracted separately by two investigators (ODV and MM), and in the event of disagreement LS cross-examined doubtful data. The following data were extracted: first author, year of publication, country of origin, study type (cross-sectional or longitudinal), study population (sample size, age, gender, period of data collection, and country of residence), type of sport/exercise, presence of a control groups, assessment method and when they were studied.

### Risk of bias

Methodological quality was assessed using [1] the Quality Assessment Tool for Observational Cohort and Cross-Sectional Studies in observational studies [2]; the Quality Assessment Tool for Before-After (Pre-Post) Studies With No Control Group in before-after (pre-post) studies. Both tools are recommended by the National Institute of Health, U.S. Department of Health and Human Services [12], which were based on Evidence-based Practice Centers (AHRQ) criteria (Additional file 1: Table S1). The [1] tool consists of 14 criteria and the [2] tool of 12 criteria that are used to assess quality, including whether the population studied was clearly specified and defined, whether the outcome assessors were blinded, and an assessment of the participation rate. The criteria were classified as “yes”, “cannot be determined”, “not reported”, or “not applicable”.

Quality rates were good, fair, or poor as judged by two independent observers (ODV and MM) following the instructions given by the National Institute of Health and taking into consideration the number of positive responses. High risk of bias translates to a rating of poor quality. Low risk of bias translates to a rating of good quality.

## Results

### Study selection

**Fig. 1:**
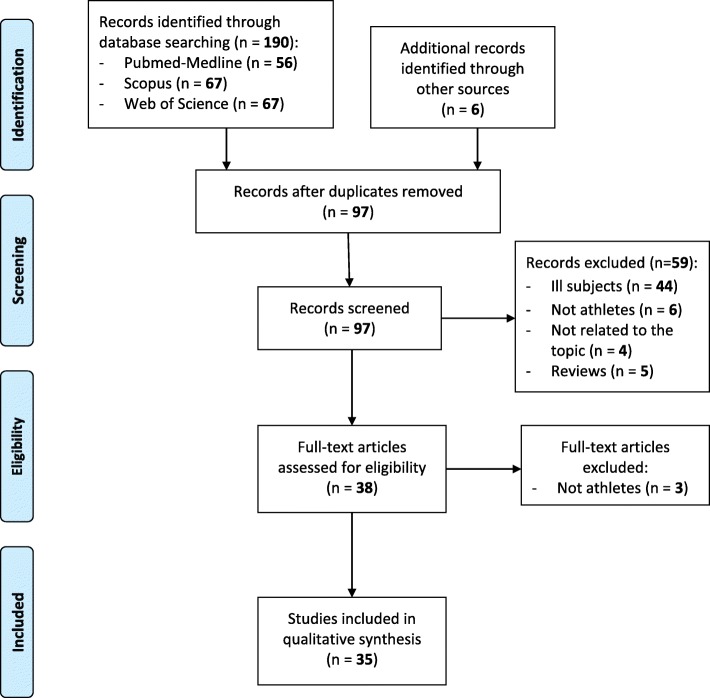
Flowchart on the search and selection of articles included in the review

The literature search revealed a total of 196 studies. After exclusion of duplicates (*n* = 99), by screening titles and abstracts 59/97 studies were excluded because included ill subjects or subjects not practicing a sport or because they were not otherwise appropriated. Five reviews were also excluded. The full text of 38 studies was independently examined by two reviewers. Thirty-five studies (21 cross-sectional and 14 longitudinal studies, of which 12 giving also cross-sectional data) meeting the inclusion criteria and being suitable for the systematic review (Fig. 1).

### Study characteristics

The main characteristics of the selected studies are summarized in Tables 1 and 2. The articles were published from 1992 to 2019 but most of them (85.7%) appeared in the last 10 years. Overall, 3703 athletes (3172 in cross-sectional and 531 in longitudinal studies) were taken into consideration in this systematic review, with more males (*n* = 2699) than females (*n* = 1264), and including children, adolescents and adults. Most of the cross-sectional studies were carried out in Europe (*n* = 14), especially in Italy (*n* = 9), six in the United States, Central or South-America and only one in Asia. All the longitudinal studies were performed in Europe (*n* = 7 in Italy, *n* = 2 Spain and Portugal, and n = 1 in France, UK and Czech Republic). Eleven studies evaluated soccer players (34.4%), eight cyclists (22.9%), six judo players (17.1%), six swimmers (17.1%), six volleyball players (17.1%), five triathlon athletes (14.3%), four water polo athletes (11.4%), four handball (11.4%) and four basketball players (11.4%). Other 31 sport specialties were considered in only one study.

**Table 1:**
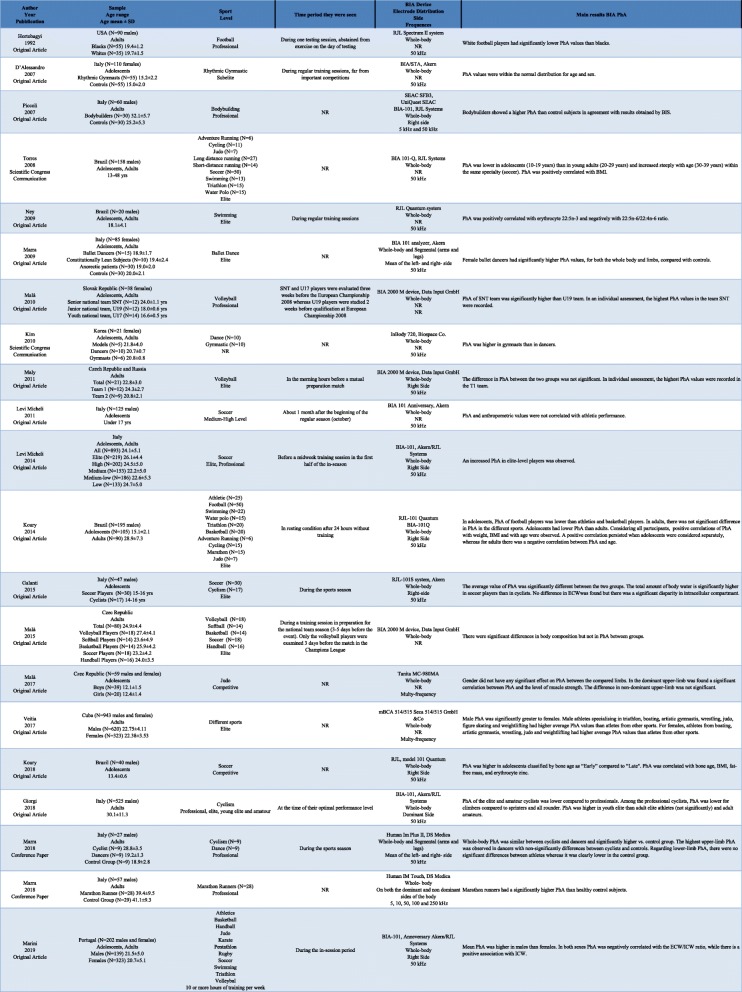
Descriptive characteristics of cross-sectional included studies (*n* = 21)

**Table 2:**
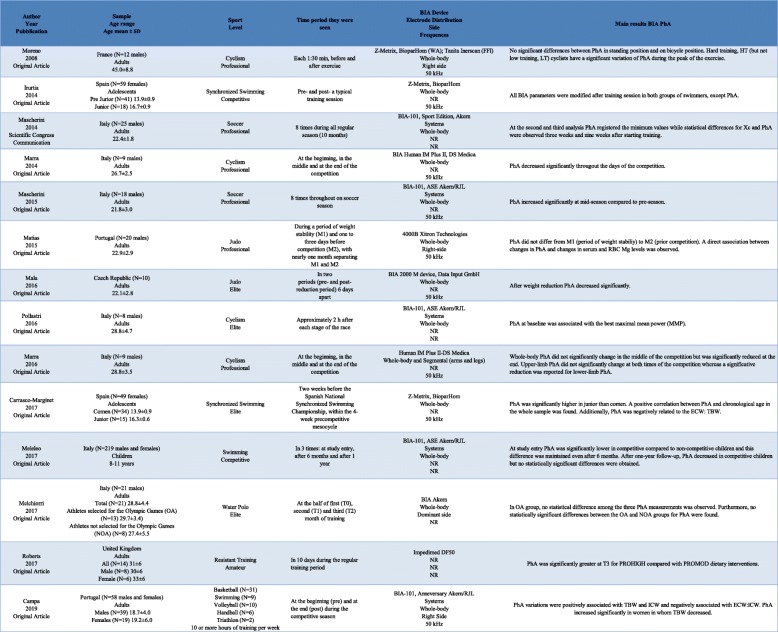
Descriptive characteristics of longitudinal included studies (*n* = 14)

Of the 35 papers analysed, 32 (91.3%) used the classic BIA, one used Tanita (2.9%), one used Inbody720 (2.9%) and one used mBCA Seca (2.9%). Piccoli et al. [13] and Matias et al. [14] measured PhA with bioelectrical impedance spectroscopy (BIS) analyser. In 29 studies phase angle was measured at 50 kHz. Piccoli et al. [13], with BIS methodology, measure PhA at 5 and 50 kHz assuming that the current path is only extracellular at the lowest frequencies and that is both extra- and intracellular at the highest frequencies. Authors in fourteen papers performed BIA and BIVA. Other information is available in in Table 1 and Table 2.

### Risk of bias

Sample size was small especially in longitudinal studies (Table 2). Measurement conditions of BIA were sometimes not completely described. Furthermore, the time period in which patients were included in the studies was not always clearly described.

The risk of overall bias was moderate to high. Three of the observational studies had an overall good rating in terms of quality, while sixteen were rated as fair and two as poor. Only two of the before-after (pre-post) studies had an overall good rating in terms of quality, eight were rated as fair and four as poor (Additional file 1: Table S1).

### Cross-sectional studies

#### Differences between athletes and controls

Six studies have compared PhA in athletes and controls.

In the paper by Piccoli et al. [13], professional male bodybuilders (*n* = 30, 31.2 ± 5.7 yrs) had a higher PhA (+ 17.8% at 50 kHz) than control subjects. This finding suggested more cell membranes per fluid volume unit, i.e. increased intracellular water and BCM.

In the same year, D’Alessandro et al. [15] found that female rhythmic gymnasts (*n* = 55, 15.2 ± 2.2 yrs) had PhA values within the normal range for age and sex. No direct comparison with a control group was reported.

Later, Marra et al. [16] showed that female ballet dancers (*n* = 15, 18.9 ± 1.7 yrs) had significantly higher PhA compared to controls, not only for the whole body (+ 9.6%) but also for upper limbs (+ 22.2%) and lower limbs (+ 10.0%).

Meleleo et al. [17] studied two groups of children: competitive individuals attending swimming and gymnastics sports clubs (*n* = 29, 8.0–10.5 yrs) vs. a control group of age-matched healthy children (*n* = 190, 8.2–10.5 yrs). At study entry PhA was significantly lower in competitive vs. non-competitive children and this difference was maintained even after 6 months. After one-year follow-up, PhA decreased in competitive children but no statistically significant differences were obtained.

Recently, Marra et al. [18] evaluated 27 young males: 9 cyclists (28.8 ± 3.5 yrs), 9 dancers (19.2 ± 1.3 yrs) and 9 young control normal-weight men (18.9 ± 2.8 yrs). Data of cyclists were collected during a three-week stage race, while dancers were studied during the ballet season. Whole-body PhA was similar between cyclists and dancers being significantly higher vs. controls (+ 11.4% and + 12.0%). The highest upper limb PhA was observed in dancers with non-significantly differences between cyclists and controls. Lower-limb PhA is similar in cyclists and dancers but lower in the control group (− 15.4%).

In another paper the same authors [19] studied 28 male marathon runners (personal best in the last year < 195 min; 39.4 ± 9.5 yrs) and 29 male control subjects with aerobic physical activity < 60 min/week. A significant difference between groups emerged (PhA + 9.7% in marathon runners).

#### Differences between genders

Differences in PhA between genders were consistently evaluated in three studies.

Veitia et al. [20] performed BIA in 943 Cuban athletes (620 males, 22.8 ± 4.1 yrs., and 323 females 22.4 ± 3.5 yrs) specialized in 26 different sports. Mean PhA value was significantly higher (+ 15.5%) in males than females, with a difference for most of the sports considered.

The same year, Mala et al. [21] assessing whole-body BIA variables in adolescent judo athletes (39 males, 12.1 ± 1.5 yrs., and 20 females, 12.4 ± 1.4 yrs) members of the Czech cadet and junior teams, observed that gender did not have a significant effect on PhA and that there was no difference between the dominant or non-dominant body sides.

Lastly, in the recent study by Marini et al. [10] on 202 athletes involved in 11 different sports mean PhA was definitely higher in males than females (+ 13.2%). No data were available for males and females practicing the same sport.

#### Differences due to age

PhA in athletes of various age were determined in five studies.

Torres et al. [22] studied 158 elite athletes (13–48 yrs) practicing adventure running, cycling, judo, long-distance running, short-distance running, soccer, swimming, triathlon and water polo. PhA was lower in adolescents (10–19 years) than in young adults (20–29 years), and increased with age within the same specialty (soccer). The highest mean value was observed in the third decade of life. In the athletes aged 10–19 years, 57% of PhA values were lower than the 5th reference percentile (6) whereas in the other three age groups the corresponding values were 2%, 0% and 0% respectively.

Mala et al. [23] evaluated PhA in three teams of female national volleyball players: a senior national team (SNT, *n* = 12, 24.0 ± 1.1 yrs), a junior national team (under 19, n = 12, 18.0 ± 0.6 yrs), and a youth national team (under 17, *n* = 14, 16.6 ± 0.5 yrs). SNT and U17 players were evaluated 3 weeks before the European Championship 2008 whereas U19 players were studied 2 weeks before qualification at European Championship 2008. The highest PhA values were recorded in the SNT group, with a significant difference between SNT and Under 19 players.

In the study by Koury et al. [24] on male adolescent (*n* = 105, 15.1 ± 2.1 yrs) and adult (*n* = 90, 28.9 ± 7.3 yrs) athletes, considering several sport groups (athletics, football, swimming, water polo, triathlon, basketball, adventure running, cycling, marathon and judo), adolescent athletes showed lower PhA than adult athletes (− 15.9%). PhA in the adolescents remained lower when sport type was used as a covariate in a multivariate general linear model (*p* < 0.001). A positive correlation between PhA and age was observed in adolescents, whereas adult athletes exhibited a negative correlation. The influence of age on PhA persisted when controlled for sport type.

More recently, Carrasco-Marginet et al. [25] evaluated young female elite synchronized swimmers of two age categories (34 comen, 13.9 ± 0.9 yrs., and 15 junior, 16.3 ± 0.6 yrs) performing a single long, high-intensity training session. They found that PhA was significantly higher in junior (+ 7.1%) than comen, with a positive correlation between PhA and age.

Finally, Giorgi et al. [26] reported that in 525 male road cyclists (30.1 ± 11.3 yrs) PhA values were higher (not significantly) in youth elite compared to adult elite athletes or adult amateurs.

#### Comparisons between different sport disciplines

Five studies compared PhA between athletes practicing different sports.

Kim et al. [27], in a conference paper, showed that PhA was higher in 6 female gymnasts (20.8 ± 0.8 yrs., PhA 5.9 ± 0.5 degrees) than 10 female dancers (20.7 ± 0.7 yrs., PhA 5.0 ± 0.3 degrees).

In the paper by Koury et al. (see above) [24], differences in PhA between various sports were evaluated. Adolescent football players had a lower mean value than track and field athletes (− 31.7%) or basketball players (− 15.3%), An overall significant difference was observed between adult athletes practicing athletics, swimming, triathlon, water polo, adventure running, cycling, marathon and judo but no pairwise comparisons were performed. Of note, sample size was small in most experimental groups (even < 10 subjects).

Galanti et al. [28] in male adolescents observed that the average value of PhA was slightly but significantly higher (7.3 ± 0.6 vs. 7.1 ± 0.5 degrees) in cyclists (*n* = 17, 14–16 yrs) than soccer players (*n* = 30, 15–16 yrs).

Mala et al. [29] studied 80 elite female players (24.9 ± 4.4 yrs) of five team sports (volleyball, softball, basketball, soccer and handball). They observed significant differences in body composition between groups (for instance, with respect to FFM), but did not detect any significant differences in PhA. The variability of PhA was high in all groups, as indicated by the large standard deviation values.

In their large study, Veitia et al. (see above) [20] studied 943 subjects which made up the Cuban adult national selection in 26 sports. In males, athletes practicing triathlon, weight lifting, boating, artistic gymnastics and wrestling had average values of PhA ≥7 degrees that were higher compared to those of other athletes. In females, athletes from boating, artistic gymnastics and weight lifting had higher average values of PhA (≥6.5 degrees) than athletes from other sports.

#### Comparisons within the same sport discipline

Three studies evaluated the possible variation of PhA due to different performance levels. Maly et al. [30] studied two volleyball teams (*n* = 12, 24.3 ± 2.7 yrs., and *n* = 9, 20.8 ± 2.1 yrs), participating in the CEV Champion League 2008–2009. The first team did not pass beyond the basic round, whereas the second one participated in the quarterfinal round. There was no significant difference in mean PhA between the two teams.

In the study by Levi Micheli et al. [31] 893 male soccer players (24.1 ± 5.1 yrs) were subdivided in five groups according to performance level (i.e. the division in which the team plays). An increased PhA was observed in the elite-level group compared to the other groups (high-level, medium-level, medium-low level and low-level).

Finally, Giorgi et al. (see above) [26] reported that PhA of elite (*n* = 79, 21.1 ± 2.9 yrs) and amateur cyclists (*n* = 232, 39.0 ± 10.5 yrs) (but not that of youth elite cyclists, *n* = 59, 16.8 ± 1.1 yrs) was lower (*p* < 0.05) in comparison to professionals (*n* = 155, 26.3 ± 4.7 yrs). Among these latter PhA was lower for climbers compared to sprinters and all-rounders (*p* < 0.05).

#### Differences due to racial and genetic profile

In the only study reporting data on racial profile, Hortobagyi et al. [32] showed that mean PhA was higher in 55 black (19.4 ± 1.2 yrs) compared to 35 white (19.7 ± 1.5 yrs) Division I American Football players.

Levi Micheli et al. [33] determined the genetic profile in a group of young adolescent Italian medium to high-level soccer players (< 17 yrs) assessing the distribution of ACE genotypes (DD, ID, II) and VDR gene (FF, Ff and ff) polymorphisms, because of their association with performance-related functions. They assessed body composition with BIA and studied the athletic performance by standard functional performance field tests (squat jump, countermovement jump, 10- and 20-m sprint time). Concerning ACE genotypes, PhA was higher in athletes harboring the D allele. Furthermore, regarding VDR gene, the FF genotype was associated with a mean PhA higher than that observed with FF and ff genotypes.

#### Correlation with other variables

Seven studies have evaluated the relationships between PhA and other variables.

In the study by Torres et al. [22] (see above) PhA was positively correlated with BMI (r = 0.66; *p* < .001). Similarly, Koury et al. (see above) [24] observed a positive association with both weight and BMI (r = 0.498 and 0.583, respectively, *p* < 0.01).

Ney et al. [34] studied 20 male short-distance swimmers (18.1 ± 4.1 yrs., 50 and 100 m freestyle) and found significant correlations of PhA with fatty acid and tocopherol composition in plasma and erythrocytes membranes. PhA was positively related (r = 0.51, *p* = 0.024) with erythrocyte 22:5 n-3 (an index of DHA deficiency). On the contrary, PhA was neither associated with other erythrocyte PUFAs, nor with indices of PUFA and DHA status, or erythrocyte tocopherols.

Levi Micheli et al. (see above) [33] claimed that in a well-trained population, PhA and anthropometric values were not correlated with athletic performance.

In the abovementioned study by Mala et al. [21] in judo adolescent athletes a significant correlation emerged between PhA and handgrip strength (boys: r = 0.64, *p* < 0.01, girls: r = 0.61, p < 0.01) for the dominant limb.

In a recent study Koury et al. [35] evaluated the relation between minerals and PhA. It was found that in 40 adolescent male soccer athletes (13.4 ± 0.6 yrs), PhA tended (*p* = 0.010) to be higher in adolescents classified by bone age as “Early” compared to “Late”. PhA also correlated (*p* < 0.05) with bone age (r = 0.562), BMI (r = 0.382), FFM (r = 0.468) and erythrocyte zinc concentration (r = 0.379). PhA was higher in adolescents with erythrocyte zinc concentration above the median than those below the median. Multiple linear regression analysis revealed that bone age (*p* = 0.001) and erythrocyte zinc concentration (*p* = 0.047) were both positive predictors of PhA.

In a relevant cross-sectional study in 202 athletes, Marini et al. [10] showed that in both males and females PhA was negatively correlated with the ECW/ICW ratio (males: r = − 0.493, *p* < 0.001; females: r = − 0.408, *p* < 0.001), while there was a positive association with ICW (males: r = 0.327, p < 0.001; females: r = 0.243, *p* = 0.080).

### Short-term studies and longitudinal studies

Only three papers evaluated changes in PhA immediately before and after a training session. In two of the three short-term studies [36, 37] there were no details regarding intensity and/or volume of the exercise session probably due to the study type (conference papers).

Moreno et al. [36] showed that in 12 male cyclists (45.0 ± 8.8 yrs) there was a non-significant difference between PhA during 30 min of exercise in standing position and on bicycle position. Hard training cyclists exhibited significant PhA changes at exercise peak, but this was not the case for the low training cyclists. Peaks correspond to maximal heart rate.

In another conference paper, junior (*n* = 18, 16.7 ± 0.9 yrs) and pre-junior (*n* = 41, 13.9 ± 0.9 yrs) female synchronized swimmers were studied by Irurtia et al. [37]. All BIA parameters, except PhA, in both groups varied after training session.

More recently, Carrasco-Marginet et al. [25] (see above) observed a significantly increased PhA between pre and post-training (*p* < 0.05) in both junior (208.4 ± 10.3 min of training with 6.8 ± 0.6 rating of perceived exertion, following the RPE scale) and comen (149.6 ± 3.3 min of training with 6.4 ± 0.5 of RPE) elite synchronized swimmers. PhA was negatively related to BIA-derived ECW/TBW ratio. No correlations were observed between bioelectrical pre to post changes in relation to BM.

Eleven papers have evaluated changes in PhA with time due to training programmes and/or other planned interventions.

Mascherini et al. [38] reported data on 11 professional male soccer players (22.4 ± 1.8 yrs) measuring their PhA eight times during regular season. Mean PhA was significantly lower than baseline 3 weeks and 9 weeks after starting training. Later, in 18 professional italian soccer players (21.8 ± 3.0 yrs) the same authors [39] found that PhA increased significantly at mid-season compared to pre-season (*p* < 0.05).

Marra et al. [40] evaluated whole-body PhA in professional cyclists (*n* = 9, 26.7 ± 2.5 yrs) participating in a 3-week stage race. They collected data at the beginning (the day before the race), halfway through (rest day, after the 9th lap) and at the end of the race (the last day, after the 20th lap). PhA was significantly decreased halfway through (Δ = − 0.51 ± 0.45, *p* < 0.001) and at the end of the race (Δ = − 1.00 ± 0.27, p < 0.001). The same authors [41] assessed segmental BIA in a group of professional cyclists (n. 9, 28.8 ± 3.5 yrs) participating in a multiple-stage bicycle race. Whole-body PhA did not significantly change after the first half of the race but significantly decreased at the end (*p* < 0.05). Upper-limb PhA did not significantly change whereas a significant reduction was reported for lower-limb PhA.

A study of Pollastri [42] on 8 elite cyclists (28.8 ± 4.7 yrs) investigated whether body water changes during a multiple-stage bicycle race affected the average maximal mean power (MMP) of different time durations. PhA at baseline was associated with the best MMP over 15 s as observed during competition (20 measurements).

Matias et al. [14] in 20 male judo athletes (22.9 ± 2.9 yrs) observed that PhA did not differ from a period of weight stability to prior competition; mean change in weight was − 0.8 ± 2.2 kg. There was a positive association between changes in PhA and those in serum and RBC Mg levels.

Meleleo et al. [17] studied two groups of children: competitive subjects attending swimming and gymnastics sports clubs (*n* = 29, 8.0–10.5 yrs) and ‘control’ age-matched healthy children (*n* = 190, 8.2–10.5 yrs). At baseline PhA was significantly lower in competitive subjects and this difference was maintained even after 6 months. After one-year follow-up, PhA decreased, but not significantly, in competitive children.

Mala et al. [43] studied 10 elite youth judo athletes (22.1 ± 2.8 yrs) before and after pre-competitive weight loss (6 day on average, using dehydration). BIA was performed before and after the weight reduction period, 6 days apart. Mean PhA significantly decreased after weight loss (− 4.1%, *p* < 0.01).

In the study of Melchiorri et al. [44], 21 male elite water polo athletes intensively trained based on a detailed program for 3 months before the Olympic Games. Only 13 athletes (OA, 29.7 ± 3.4 yrs) participated to the Olympics Games, 8 were excluded (NOA, 27.4 ± 5.5 yrs). PhA was evaluated after the first (T0), second (T1) and third (T2) month of training. There was no statistical difference among the three measurements for PhA in the OA group. Furthermore, data showed no statistically significant differences of PhA between the OA and NOA groups.

Campa et al. [45] performed BIA in 58 athletes at baseline and after 6 months during the competitive season. PhA variations were positively associated with TBW and ICW and negatively associated with the ECW/ICW ratio.

Finally, although not concerning athletes, we considered Roberts’s study [46] because it gave some information on the effects of protein supplementation plus physical activity on phase angle. This is an interesting topic for future research on sport nutrition. The authors studied the effects of a resistance training programme (at least 3 h/week) in 14 resistance-trained individuals (8 males 30 ± 6 yrs.; 6 females 33 ± 6 yrs). They underwent two 10-day isocaloric dietary regimes with a protein content of 1.8 g × kg^− 1^ × d^− 1^ (PROMOD) or 2.9 g × kg^− 1^ × d^− 1^ (PROHIGH). On days 8–10 (T1-T3), participants undertook resistance exercise under controlled conditions, performing 3 sets of squats, bench press and bent-over rows at 80% 1 repetition maximum until volitional exhaustion. In PROHIGH group PhA increased at T3 compared to T1 and T2, while it tended to decrease in the PROHIGH group, although not significantly. PhA was slightly higher at T3 for PROHIGH (+ 2.2%) compared with PROMOD (*p* = 0.012).

## Discussion

BIA is applied in athletes as a field technique to estimate body composition, being useful in sport science for single measurements or for tracking body composition changes [7]. On the other hand, raw BIA variables, such as PhA or IR, are commonly related to ECW/ICW ratio, BCM, and cellular integrity [2]. In addition, an association between muscle strength and PhA has been observed in various pathophysiological conditions (for instance,1–3), suggesting that raw BIA may be useful in assessing muscle quality.

In this context, only a few papers have so far evaluated raw BIA variables in athletes. A recent systematic review examined the applications of BIVA in sports and exercise, a methodology giving information on hydration status by analyzing the length of bioimpedance vector and its inclination [9]. The authors concluded that the current technique, called “classical BIVA”, is not fully reliable to identify dehydration in individual athletes. The review by Custodio Martins et al. [47] explored the use of different BIA-derived estimates of body composition in athletes, adding a concise, preliminary view on PhA, a raw BIA variable that has been considered in recent years for assessing body composition in various pathophysiological conditions [1–3].

In this systematic review, we aimed to extend previous information on PhA values as measured in athletes by focusing in depth on different issues of interest. Thirty-five papers were selected according to inclusion and exclusion criteria. In almost all cases single-frequency BIA has been performed (on the whole body). Although it is well known the standardization of measurement conditions is essential for obtaining accurate and reproducible BIA data, most of selected studies did not give enough details in this respect, in particular on the length of time since the last training session (a critical aspect especially in the case of strenuous exercise).

One might expect that training, especially muscle strengthening, should affect not only muscle function but also BCM and muscle cell mass. The first question in this study sought to determine whether PhA differs between athletes and control subjects. Surprisingly, only few papers have so far addressed this issue, sometimes in small groups of athletes. A very marked increase in PhA was observed in bodybuilders [13] (+ 17.8% on the average), female dancers [16] (+ 9.6%), male dancers [18] (+ 12.0%), cyclists [18] (+ 11.4%) and marathon runners [19] (+ 9.7%).

Thus, these findings suggest that muscle strengthening causes a greater increase in PhA compared to endurance training. Indeed, contrary to expectations, Meleleo et al. [17] reported that PhA was significantly lower in competitive vs. non-competitive children, suggesting that the effects of training on PhA may be different in childhood.

As far as main individual’s characteristics are concerned, in the general population PhA increases with age in both genders until late adulthood and then decrease in the elderly [22–26], with a between-gender difference that becomes greater through adolescence [48, 49] and with mean values in adult age consistently higher in males than females [5, 6].

The papers selected for gender diversity are in line with the aforementioned findings, with no difference in young adolescent judo athletes [21] and significant higher values in adolescent/adult male compared to female athletes [20]. Similarly, four out of five selected papers reported an age trend in various sports [22–25], whereas a single paper found the opposite, with higher PhA in adolescent male than adult male road cyclists [26]. It should be noted that differences in years of practice and training programmes may influence changes with time.

A key point of the present review was to evaluate whether and to what extent PhA differs between different sports and performance levels. Overall, the selected papers have provided inconsistent and puzzling findings, possibly because of inappropriate study design (for instance, in selecting subjects) or small sample sizes. The variability of PhA was high, as indicated by large standard deviation values [27–29]. Variations between sports emerge but no definite conclusions could be drawn on endurance vs. resistance training or recreational vs. competitive sports, although some results suggest indirectly that PhA increase with muscle-strengthening activities [20].

Turning to athletes of the same sport, two studies [26, 31] demonstrated that PhA was higher in soccer players and cyclists with a better performance level, whereas another one did not find differences between a stronger and a weaker volleyball teams [30]. Thus, it could be argued (but not definitely demonstrated), that the relationships between PhA and performance level may vary in different sports and are possibly influenced by the criteria used to assess performance level. Interestingly, changes emerge also for the same sport when athletes differ depending on their physical characteristics. For instance, among cyclists PhA was lower for climbers compared to sprinters and all-rounders [26].

Overall, in order to interpret variability of PhA, a single study [33] indicated that PhA is influenced by ACE or VDR gene polymorphisms, in line with their involvement in a variety of performance-related functions. In addition, another study has shown that mean PhA was higher in white than black football players [32], which may not surprising given that differences in body composition due to ethnicity are well known [50].

Finally, longitudinal evaluation of body composition may offer, at least in theory, relevant information on the changes in body composition and hydration due to training or untraining, which might be associated with physical performance. Unfortunately, the papers selected for the present review [14, 17, 25, 36–46] have given inconsistent results. A comprehensive view of the issue cannot be formed because they considered different athletic disciplines and had very different experimental protocols (sometimes with small experimental groups).

## Conclusions

This systematic review aimed to summarise the current knowledge on the evaluation of BIA-derived PhA in athletes. Of note, two recent studies strongly support the idea that PhA is an index of ECW/ICW ratio or BCM [10, 45]. PhA increases with age and is likely to be higher in males. Unfortunately, it is still uncertain to what extent PhA varies between different sports and changes with training/untraining. It can be argued that for a given sport much more data should be collected in a systematic way and for a period of time appropriate in order to determine changes and trends. This is even more crucial in the case of intervention studies.

From a practical point of view, at the present time the measurement of PhA is a promising approach to evaluate muscle quality in groups of athletes, for instance detrained compared to well-trained subjects. On the other hand, further studies are needed to specify the most appropriate measurement conditions and to assess to what extent PhA may be a reliable index for identifying individual’s characteristics critical to performance, evaluating the effects of training programs, managing weight strategies in weight-category sports, etc.

## Supplementary information

**Additional file 1:** **Table S1.** Quality Assessment Tool for Observational Cohort and Cross-Sectional Studies. **Table S2.** Quality Assessment Tool for Observational Cohort and Cross-Sectional Studies (Longitudinal Studies). **Table S3.** Quality Assessment Tool for Before-After (Pre-Post) Studies With No Control Group. Risk of bias scores of included studies.

## Data Availability

All data pertaining to the conclusions of the study are found within the article. The corresponding data set used is available under reasonable requests.

## Abbreviations

BCM: Body Cell Mass
BIA: Bioelectrical Impedance Analysis
BIS: Bioelectrical Impedance Spectroscopy
BIVA: Bioelectrical Impedance Vector Analysis
ECW: Extracellular Water
FFM: Fat-Free Mass
FM: Fat Mass
ICW: Intracellular Water
IR: Impedance Ratio
PhA: Phase Angle
R: Resistance
TBW: Total Body Water
Xc: Reactance
Z: Impedance
