# Supplementary material for: Bioelectrical impedance phase angle in sport: a systematic review
Source: J Int Soc Sports Nutr. 2019 Nov 6;16:49. doi: 10.1186/s12970-019-0319-2 (PMC6833254; doi:10.1186/s12970-019-0319-2)
Supplement: Supplementary file 1 — Additional file 1: Table S1. Quality Assessment Tool for Observational Cohort and Cross-Sectional Studies. Table S2. Quality Assessment Tool for Observational Cohort and Cross-Sectional Studies (Longitudinal Studies). Table S3. Quality Assessment Tool for Before-After (Pre-Post) Studies With No Control Group. Risk of bias scores of included studies. [file 12970_2019_319_MOESM1_ESM.docx]

**Table S1**. Quality Assessment Tool for Observational Cohort and Cross-Sectional Studies

| **Criteria** | **Hortobagyi 1992** | **D’Alessandro 2007** | **Piccoli 2007** | **Torres 2008** | **Ney 2009** | **Marra 2009** | **Malà 2010** | **Kim 2 010** | **Maly’2011** | **Levi Micheli 2011** | **Levi Micheli 2014** | **Koury 2014** | **Galanti 2015** | **Malà 2015** | **Malà 2017** | **Veitia 2017** | **Koury 2018** | **Giorgi 2018** | **Marra 2018** | **Marra 2018** | **Marini 2019** |
| --- | --- | --- | --- | --- | --- | --- | --- | --- | --- | --- | --- | --- | --- | --- | --- | --- | --- | --- | --- | --- | --- |
| 1. Was the research question or objective in this paper clearly stated? | yes | yes | yes | yes | yes | yes | yes | no | yes | yes | yes | yes | no | yes | yes | yes | yes | yes | yes | yes | yes |
| 2. Was the study population clearly specified and defined? | yes | yes | yes | yes | yes | yes | yes | no | yes | yes | yes | yes | no | yes | yes | yes | yes | yes | yes | yes | yes |
| 3. Was the participation rate of eligible persons at least 50%? | unc | unc | unc | unc | unc | yes | unc | unc | unc | unc | unc | no | unc | unc | unc | unc | unc | unc | unc | unc | unc |
| 4. Were all the subjects selected or recruited from the same or similar populations (including the same time period)? Were inclusion and exclusion criteria for being in the study prespecified and applied uniformly to all participants? | yes | yes | no | no | na | yes | no | yes | yes | na | yes | no | unc | yes | yes | no | no | no | no | no | yes |
| 5. Was a sample size justification, power description, or variance and effect estimates provided? | no | no | no | no | no | no | no | unc | no | no | no | no | no | no | no | no | no | no | no | na | no |
| 6. For the analyses in this paper, were the exposure(s) of interest measured prior to the outcome(s) being measured? | na | no | no | na | na | no | no | na | na | na | na | na | no | na | no | yes | na | na | na | na | na |
| 7. Was the timeframe sufficient so that one could reasonably expect to see an association between exposure and outcome if it existed? | na | na | na | na | na | no | no | na | na | na | na | na | na | na | no | yes | na | na | na | na | na |
| 8. For exposures that can vary in amount or level, did the study examine different levels of the exposure as related to the outcome (e.g., categories of exposure, or exposure measured as continuous variable)? | na | na | na | na | na | na | na | na | na | na | na | na | no | na | na | unc | na | na | na | na | na |
| 9. Were the exposure measures (independent variables) clearly defined, valid, reliable, and implemented consistently across all study participants? | na | na | na | no | na | yes | na | no | na | na | na | na | no | na | no | na | na | na | no | no | na |
| 10. Was the exposure(s) assessed more than once over time? | no | no | no | no | no | no | no | no | no | no | no | no | no | no | na | no | no | no | no | no | no |
| 11. Were the outcome measures (dependent variables) clearly defined, valid, reliable, and implemented consistently across all study participants? | yes | yes | yes | no | yes | yes | yes | no | yes | yes | yes | na | no | yes | yes | yes | yes | na | no | no | yes |
| 12. Were the outcome assessors blinded to the exposure status of participants? | unc | unc | unc | unc | unc | unc | unc | unc | unc | unc | unc | na | no | unc | unc | unc | unc | unc | unc | unc | unc |
| 13. Was loss to follow-up after baseline 20% or less? | na | na | na | na | na | na | na | na | na | na | na | na | na | na | na | na | na | na | na | na | na |
| 14. Were key potential confounding variables measured and adjusted statistically for their impact on the relationship between exposure(s) and outcome(s)? | na | na | na | na | na | no | na | na | na | na | na | na | na | na | na | na | na | na | na | na | na |
| **Quality Rating (Good, Fair, or Poor)** | **Good** | **Fair** | **Fair** | **Fair** | **Fair** | **Good** | **Fair** | **Poor** | **Fair** | **Fair** | **Fair** | **Fair** | **Poor** | **Fair** | **Fair** | **Fair** | **Fair** | **Fair** | **Fair** | **Fair** | **Good** |
| Comments (If POOR, please state why): |  |  |  |  |  |  |  | Low n° of participants |  |  |  |  | Methodologies are not appropriate |  |  |  |  |  |  |  |  |

Rater #1 Initials: ODV; Rater #2 Initials: MM.

Legend: the items were classified as yes; no; unclear (unc) or not applicable (na). Then the Quality Rating of each study was rated **Good**; **Fair** or **Poor**

**Table S2.** Quality Assessment Tool for Observational Cohort and Cross-Sectional Studies (Longitudinal Studies)

| **Criteria** | **Moreno 2008** | **Irurtia 2014** | **Mascherini 2014** | **Marra 2014** | **Mascherini 2015** | **Matias 2015** | **Malà 2016** | **Marra 2016** | **Carrasco-Marginet 2017** | **Melchiorri 2017** | **Roberts 2017** |
| --- | --- | --- | --- | --- | --- | --- | --- | --- | --- | --- | --- |
| 1. Was the research question or objective in this paper clearly stated? | yes | yes | yes | yes | yes | yes | yes | yes | yes | yes | yes |
| 2. Was the study population clearly specified and defined? | yes | yes | yes | yes | yes | yes | yes | yes | yes | yes | yes |
| 3. Was the participation rate of eligible persons at least 50%? | unc | unc | unc | unc | unc | unc | unc | unc | unc | unc | unc |
| 4. Were all the subjects selected or recruited from the same or similar populations (including the same time period)? Were inclusion and exclusion criteria for being in the study prespecified and applied uniformly to all participants? | na | no | yes | yes | yes | yes | yes | yes | yes | yes | no |
| 5. Was a sample size justification, power description, or variance and effect estimates provided? | no | no | no | no | no | no | no | no | no | no | no |
| 6. For the analyses in this paper, were the exposure(s) of interest measured prior to the outcome(s) being measured? | na | na | na | na | na | na | na | na | na | na | na |
| 7. Was the timeframe sufficient so that one could reasonably expect to see an association between exposure and outcome if it existed? | na | no | yes | yes | yes | no | no | yes | yes | yes | no |
| 8. For exposures that can vary in amount or level, did the study examine different levels of the exposure as related to the outcome (e.g., categories of exposure, or exposure measured as continuous variable)? | na | no | na | na | na | na | no | na | na | na | na |
| 9. Were the exposure measures (independent variables) clearly defined, valid, reliable, and implemented consistently across all study participants? | na | na | na | na | na | na | na | na | na | na | na |
| 10. Was the exposure(s) assessed more than once over time? | no | yes | yes | yes | yes | yes | yes | yes | yes | yes | yes |
| 11. Were the outcome measures (dependent variables) clearly defined, valid, reliable, and implemented consistently across all study participants? | no | no | yes | yes | yes | yes | no | yes | yes | yes | yes |
| 12. Were the outcome assessors blinded to the exposure status of participants? | unc | unc | unc | unc | unc | un | unc | un | unc | un | un |
| 13. Was loss to follow-up after baseline 20% or less? | na | na | na | na | na | na | na | na | na | na | na |
| 14. Were key potential confounding variables measured and adjusted statistically for their impact on the relationship between exposure(s) and outcome(s)? | na | na | na | na | na | na | na | na | na | na | na |
| **Quality Rating (Good, Fair, or Poor)** | **Poor** | **Poor** | **Fair** | **Fair** | **Fair** | **Poor** | **Poor** | **Good** | **Fair** | **Fair** | **Fair** |
| Comments (If POOR, please state why): | Outcomes not properly definited | Methodologies are not appropriate |  |  |  | Methodologies are not appropriate | Results are not clearly definited |  |  |  |  |

Rater #1 Initials: ODV; Rater #2 Initials: MM.

Legend: the items were classified as yes; no; unclear (unc) or not applicable (na). Then the Quality Rating of each study was rated **Good**; **Fair** or **Poor**

**Table 3.** Quality Assessment Tool for Before-After (Pre-Post) Studies With No Control Group

| **Criteria** | **Moreno 2008** | **Irurtia 2014** | **Malà 2016** | **Carrasco-Marginet 2017** | **Mascherini 2014** | **Mascherini 2015** | **Marra 2014** | **Marra 2016** | **Meleleo 2017** | **Melchiorri 2017** | **Roberts 2017** | **Pollastri 2016** | **Matias 2015** | **Campa 2019** |
| --- | --- | --- | --- | --- | --- | --- | --- | --- | --- | --- | --- | --- | --- | --- |
| 1. Was the study question or objective clearly stated? | yes | yes | Yes | yes | yes | yes | yes | yes | yes | yes | yes | yes | yes | yes |
| 2. Were eligibility/selection criteria for the study population prespecified and clearly described? | yes | yes | yes | yes | yes | yes | yes | yes | yes | yes | yes | yes | yes | yes |
| 3. Were the participants in the study representative of those who would be eligible for the test/service/intervention in the general or clinical population of interest? | yes | yes | Yes | yes | yes | yes | yes | yes | yes | yes | yes | yes | yes | yes |
| 4. Were all eligible participants that met the prespecified entry criteria enrolled? | yes | yes | unc | yes | unc | unc | yes | yes | unc | unc | unc | unc | unc | yes |
| 5. Was the sample size sufficiently large to provide confidence in the findings? | unc | unc | unc | unc | unc | unc | unc | unc | unc | unc | unc | unc | unc | unc |
| 6. Was the test/service/intervention clearly described and delivered consistently across the study population? | no | yes | yes | yes | yes | yes | yes | yes | yes | yes | yes | yes | unc | yes |
| 7. Were the outcome measures prespecified, clearly defined, valid, reliable, and assessed consistently across all study participants? | no | no | no | yes | yes | yes | yes | yes | yes | yes | yes | yes | no | yes |
| 8. Were the people assessing the outcomes blinded to the participants' exposures/interventions? | unc | unc | unc | unc | unc | unc | unc | unc | unc | unc | unc | unc | unc | unc |
| 9. Was the loss to follow-up after baseline 20% or less? Were those lost to follow-up accounted for in the analysis? | na | na | na | na | na | na | na | na | na | na | na | na | na | na |
| 10. Did the statistical methods examine changes in outcome measures from before to after the intervention? Were statistical tests done that provided p values for the pre-to-post changes? | yes | yes | yes | yes | yes | yes | yes | yes | yes | yes | yes | yes | yes | yes |
| 11. Were outcome measures of interest taken multiple times before the intervention and multiple times after the intervention (i.e., did they use an interrupted time-series design)? | yes | no | no | no | yes | yes | yes | yes | yes | yes | no | yes | no | yes |
| 12. If the intervention was conducted at a group level (e.g., a whole hospital, a community, etc.) did the statistical analysis take into account the use of individual-level data to determine effects at the group level? | unc | unc | unc | unc | unc | unc | unc | unc | unc | unc | unc | unc | unc | unc |
| **Quality Rating (Good, Fair, or Poor)** | **Poor** | **Poor** | **Poor** | **Fair** | **Fair** | **Fair** | **Fair** | **Good** | **Fair** | **Fair** | **Fair** | **Fair** | **Poor** | **Good** |
| Comments (If POOR, please state why): | Outcomes not properly definited | Methodologies are not appropriate | Results are not clearly definited |  |  |  |  |  |  |  |  |  | Methodologies are not appropriate |  |

Rater #1 Initials: ODV; Rater #2 Initials: MM.

Legend: the items were classified as yes; no; unclear (unc) or not applicable (na). Then the Quality Rating of each study was rated **Good**; **Fair** or **Poor**
